# Peer relations scale for adolescents treated for substance use disorder: a factor analytic presentation

**DOI:** 10.1186/1747-597X-7-29

**Published:** 2012-07-12

**Authors:** Ping Yao, James R Ciesla, Kathryn D Mazurek, Sherilynn F Spear

**Affiliations:** 1 Public Health and Health Education Programs, Northern Illinois University, DeKalb, IL, 60115, USA

**Keywords:** Substance abuse treatment, Psychoactive substance use disorder, Adolescents, Treatment outcomes, Social support, Relapse

## Abstract

**Background:**

The literature indicates that peer relations are an important aspect of the treatment and recovery of adolescents with substance use disorder (SUD). Unfortunately, no standard measure of peer relations exists. The objective of this research is to use exploratory factor analysis to examine the underlying factor structure of a 14-item peer relations scale for use in this treatment population.

**Methods:**

Participants are 509 adolescents discharged from primary substance abuse treatment from 2003–2010. The data are from research conducted between six and twelve months post discharge via a 230-item questionnaire that included the 14-item peer relations scale. The scale has questions that assess the degree to which the adolescent's social contacts conform to norms of positive behavior and therefore foster non-use and recovery. The response rate was 62%.

**Results:**

The scale was decomposed by principal component factor analysis. When the matrix was rotated by varimax a three factor solution explaining 99.99% of the common variance emerged. The first factor yielded ten items that measure association with peers who engage in positive versus delinquent social behavior (positive versus negative social behavior). The three items in the second factor specify association with peers who use versus those who don’t use drugs, and thereby encourage recovery and discourage drug use (drug use). The third and factor contained two items measuring the degree to which the recovering adolescent associates with new or previous friends (post treatment peer association).

**Conclusions:**

This scale is useful as a standard measure in that it begins to identify the measurable dimensions of peer relations that influence sustaining post treatment recovery.

## Background

The adolescent substance abuse treatment literature is replete with evidence supporting the fact that the relationships adolescents maintain with their friends and other social contacts are of great importance in understanding substance use initiation, persistence, abuse, addiction, treatment for addiction, recovery from addiction, and relapse to use after treatment [1-4]. Despite this wide-ranging literature, researchers are still faced with a twofold challenge. The first challenge is to specify the components of social support that have the most significant influence on an adolescent’s sustaining post-treatment recovery. This is important since theory grounded research has long recognized that the influence of the variables embedded in the social support construct differ in the processes of initiating versus sustaining behavior [5-7]. The second challenge is to determine how to specify the “behavior sustaining variables” in easily measurable behavioral terms. To effectively make the link between research and practice, treatment providers need to better able design interventions that target specific behaviors.

The construct “peer relations” is embedded in the broader construct of social support [8]. A variety of definitions may be used to describe social support, but most consistently, the definition is the individual’s belief that they are loved, cared for, valued and involved in a network of reciprocal commitment and responsibility [9,10]. In this context, peer relations are social relationships and interpersonal interaction processes that take place between peers and are related to sustained abstinence from drug use [5]. Social support is related to self-efficacy and outcome expectancy which are based in social learning theory [11]. Self-efficacy can describe the belief that a person has the ability to regulate and modify behavior [11]. Applications of social support relate it to a stress buffering model that has been applied to various aspects of adolescent health [12] and that could be a useful theoretical context for research on adolescent substance abuse treatment. In particular, peer-supportive communities are known to be beneficial and have improved patient outcomes [13,14].

Several papers have applied the constructs of peer relations and social support to the field of adolescent substance abuse treatment. For example, in their review of substance use treatment outcomes among adolescents, Williams and Chang [15] noted that peer social support is an important post treatment variable related to positive treatment outcome. Boisvert, Martin, Grosek & Clarie [16] found significant reductions of risk of relapse in clients who participated in peer-supported community programs. Also, Azrin and colleagues found that a new behavioral treatment focusing on restructuring family and peer relations was superior to a supportive counseling program [17].

Adolescents' peer characteristics may enhance or decrease the potential for risky behaviors [18]. For example, low levels of peer substance use during follow-up are consistently associated with better treatment outcomes among adolescents [19]. In a recent survey of 102 urban adolescents enrolled in a substance abuse treatment program, Mason [20] identified peer network characteristics that were associated with adolescent substance use and non-use: presence of daily substance users in network, engagement in negative activities, presence of peers who support non-substance use, engagement in positive activities, and presence of no daily substance users in network.

Unfortunately, as is true with so many other aspects of the adolescent substance abuse treatment literature, there are no standards for operationalizing, measuring and analyzing the peer relations construct. An exhaustive literature search turned-up no established measures of peer relations published in the academic and related literature. Therefore, any relationship between peer relations to theoretical models that could inform research and practice by placing the construct in a broader theoretical context is unknown.

The purpose of this research is: (1) to show the underlying factor structure of a scale designed to measures the peer relations of adolescents treated for substance use disorder by using exploratory factor analysis, (2) to suggest a possible tie-in of the construct of peer relationships to the literature on social support and (3) to discuss the policy implications and practical relevance of the scale to the treatment community. If it can be shown that peer relations are protective of relapse and the construct is defined and operationalized in a manner appropriate for adolescent substance abuse population, then the academic and treatment communities can improve and design treatment programs consistent with the research. Once a targeted measure of peer relations is developed and becomes available to the treatment and research communities, it will be much easier to design and evaluate treatment programs based on the broad theoretical guidance of social learning theory.

## Methods

### Participants

The participants in this study are adolescents discharged from a primary substance abuse treatment facility in the Midwest region of the United States from the eight successive years 2003–2010 (n = 509). The data were obtained from a survey of patient outcomes the facility sponsors annually. The outcomes study is conducted by independent, university-based researchers. The response rate was 62%.

The outcomes study begins each year in January when the researchers obtain a list of all of the adolescents who successfully completed treatment in the previous year and who have given appropriate consent. The sampling frame is all successful discharges in the previous year. The treatment facility discharges adolescents “with staff approval” if the adolescent has met all of his or her treatment goals which include maintaining abstinence from drugs and alcohol during treatment and a host of other behavioral goals. The criteria for treatment success comport with prevailing professional standards in the substance abuse treatment field.

Each adolescent is contacted via telephone and asked to complete a 230-item questionnaire that contains questions in several domains thought to be related to treatment success, including: school/work, family, friends, criminal behavior, and relapse. The14-item peer support scale was embedded in this questionnaire.

The list of consenting adolescents (the call list) contains the names and telephone numbers of the adolescent’s parent(s), guardian or guardian *ad litem,* emergency contact number and other contact information (grandparents, cell phone numbers, places of employment, etc.). Great effort is made to track-down and contact the adolescents; telephone numbers that are disconnected are recalled at a later date in case the number was reconnected, directories of telephone numbers are used to locate the adolescents and the treatment facility is queried for updated information. The interviews were conducted at prearranged times so the privacy of the answers could be assured (e.g. when parents were away or out of earshot). Every reasonable attempt is made to contact the adolescents but as is the case in all survey research, some of the adolescents could not be contacted and were thus lost to follow-up. Recently published research based on the data used in this study showed that characteristics of the responders and nonresponders are similar across a wide range of variables thought to predispose relapse; these findings indicate that loss to follow-up did not introduce response bias and that the missing cases can be considered missing at random [21].

Only adolescents who agreed to participate by giving consent (assent where appropriate) were contacted. At the time of admission, the adolescent and their parents are asked to give consent/assent to participate in the outcomes study. The consent/assent includes permission to contact them via telephone sometime after they are discharged and to release their treatment information to the researchers. The potential participants are assured that the researchers are bound by federal confidentiality and privacy regulations. The research protocol, including the consent process, was reviewed by the first author’s university institutional review board. None of the adolescents chose not to participate.

The treatment facility uses standard methods in assessing incoming adolescents and in making level-of-care placements. First, the Diagnostic and Statistical Manual of Mental Disorders fourth edition (DSM-IV) is used to assign diagnosis. Second, the American Society of Addiction Medicine (ASAM), Patient Placement Criteria (PPC-II) is used to place patients in appropriate levels of care. All participants in this study met the DSM criteria for dependence or abuse and were assigned to the ASAM Level I.A (primary inpatient treatment).

### Data analysis

The analysis was generated using SAS® 9.2 software [21]. First, basic descriptive and summary statistics were run in order to determine the completeness of the responses to the questions in the peer relations scale and the suitability of the data for factor analysis. Second, the factorability of the scale was evaluated by Kaiser-Meyer-Olkin measure of sampling adequacy, Bartlett’s test of sphericity, anti-image correlation analysis, and communality variation. Third, principle component analysis (PCA) was performed to identify and compute composite peer relations scores for the factors underlying the construct peer relations. Fourth, the matrix was rotated orthogonally using the varimax procedure in order to render the final factor matrix. All factor analytical procedures were performed using the SAS® 9.2 *Proc Factor* procedure [22].

## Results

### Data summary

As shown in Table 1, the sample was predominantly under 18 years of age with the average age being 17.34 (S.D. 1.55) and no one under age 12 or over age 21. Males outnumbered females 65.0% to 35.0%, respectively. Most of the sample was Caucasian (86.5%) with African-Americans and Hispanics the second and third most numerous with 6.5 and 5.8%, respectively. Marijuana usage was reported the primary substance of abuse by 55.4% of the sample, with alcohol reported by 29.7%. Cocaine, amphetamines and opiates were reported less frequently with 8.4, 3.4 and 2.0%, respectively.

**Table 1:** Demographic Characteristics of the Sample (N = 509)

**Characteristic**	**Percent**	**Number**
**Age**^**1**^		
**< 18 years**	52.0	265
**≥ 18 years**	48.0	244
**Gender**	65.0	331
	35.0	178
**Race**		
**African-American**	6.5	33
**Caucasian**	86.6	438
**Hispanic**	5.8	29
**Asian/PI**	0.8	4
**Other**	0.3	2
**Primary Drug of Dependence**		
**Alcohol**	29.7	151
**Amphetamines**	3.4	17
**Marijuana**	55.4	283
**Cocaine**	8.4	42
**Opiates**	2.0	10
**Other Drug**	1.1	6

^1^Mean = 17.34, S.D. 1.55, Range 12-21.

### Factor analysis

A total of 13 of the 14 items correlated at the 0.31 level with at least one other item, suggesting reasonable factorability. The Kaiser-Meyer-Olkin measure of sampling adequacy was 0.727, above the recommended value of 0.6 [23], and Bartlett’s test of sphericity was significant, χ^2^(91) =1066.89, p<0.0001. The diagonals of the anti-image correlation matrix were all over 0.57, supporting the inclusion of each item in the factor analysis. Finally, the communalities were all above 0.1 except for item 6 “get into arguments/fights?” (See Table 2), further confirming that each item shared some common variance with other items. Given this evidence of factorability, factor analysis was conducted with all 14 items.

**Table 2:** Factor analysis with varimax orthogonal rotation for 14 items from peer relations scale (N = 509)

**How many of your friends**^**1**^**. . .**	**Item No.**	**Mean**	**SD**	**Min.-Max.**	**Factor 1**^**2**^	**Factor 2**	**Factor 3**	**Communality**
**are new friends since treatment?**	4	1.97	1.00	1-5			0.838	0.733
**are same friends as before treatment?**	3	2.89	1.29	1-5			0.834	0.744
**attend school regularly?**	5	2.91	1.60	1-5	0.211			0.102
**hang out with "gangs"?**	10	3.36	1.55	1-5	0.331			0.116
**get into arguments/fights?**	6	3.67	1.25	1-5	0.239			0.096
**spend time with their families?**	7	4.05	1.12	1-5	0.340			0.165
**have your parents met your friends?**	1	4.38	0.85	1-5	0.369			0.141
**go to jail or prison?**	11	3.47	1.15	1-5	0.420			0.210
**do your parents like and approve of?**	2	3.57	1.12	1-5	0.521			0.311
**use drugs?**	9	4.02	0.83	1-5	0.510	0.508		0.556
**drink too much alcohol?**	8	3.79	0.99	1-5	0.608			0.440
**cause trouble for you?**	12	4.36	1.09	1-5	0.474			0.246
**encourage you to stay in treatment?**	13	4.34	1.41	1-5		0.728		0.548
**help you quit drugs?**	14	3.69	1.83	1-5		0.739		0.577
**Cronbach Alpha**					0.71	0.80	0.87	
**Eigenvalues**					1.87	1.61	1.51	
**Percent of common variance**					37.47	32.26	30.26	
**Percent of total variance**					13.36	11.50	10.79	
**Number of measures**					10	3	2	

^1^1 = never, 2 = rarely, 3 = sometimes, 4 = often, 5 = almost always.

^2^ factor loadings < 0.20 are suppressed.

Principle component analysis results show that three factors are retained by the proportion criterion setting [24]. The final total community estimate is 4.99, of which common variance explained by factor 1 is 3.13 (62.73%), factor 2 is 1.10 (22.04%), and factor 3 is 0.75 (15.03%). The total variance explained by factors 1, 2 & 3 are 22.36%, 7.86% and 5.36%, respectively.

The results of a varimax rotation of the solution are shown in Table 2. When loadings less than 0.20 were excluded, the analysis [25] yielded a three-factor solution with a simple structure that explained 35.64% of the variance. This solution was preferred because the factors appear to create item groupings that are clinically relevant and that may operationalize the peer relations construct. The common variance explained by factor 1 is 1.87 (37.47%), by factor 2 is 1.61 (32.26%), and by factor 3 is 1.51 (30.26%). The total variance explained by factor 1, 2, 3 are 13.36%, 11.5%, 10.79%, respectively.

Ten items loaded onto the first factor (Factor 1), positive versus negative social behavior, and it is clear from Table 2 that these ten items are attributes of friends that indicate the friend’s potential to be a negative or positive influence. This factor can be considered a non differentiated factor as it relates to the content areas of peer relations.

Three items load onto a second factor (Factor 2), drug use. This factor clearly represents the peer relations content area relating to emotional support. Items 13 and 14 are aspects of encouragement indicating the caring component implicit in emotional aspects of peer relations. While item 9, “uses drugs” is also part of Factor 1, it stands to reason that emotional support of drug and alcohol abstinence best comes from those who do not use drugs or alcohol.

The two items that loaded onto the third factor (Factor 3), post treatment peer association, indicate the degree to which the adolescent was able to establish new friendships. Changing peer relations shows self-efficacy which is a common emphasis in many treatment approaches [3]. Choosing to associate with new, non-using friends places the adolescents in the position to have positive appraisals from friends, an aspect of peer relations best provided by non-using friends. The two items comprising this factor have very strong loadings, .834 and .838 for items 3 and 4 respectively indicating that this factor is clearly independent.

## Discussion

Other aspects of the psychometric properties of this scale have been published elsewhere in the literature. For example, using Rasch analysis, Ciesla [26] found that the scale has many desirable characteristics. The person reliability and the Cronbach's alpha person raw score reliability both indicate that the scale is a strong metric. The item reliability is high and shows that the model is reliable. The real separation shows that the scale items are placed reliably on the Rasch "ruler" with about eight levels of importance identified. The mean-square statistic of the infit and outfit values indicated a low level of randomness and thus unidimensionality of the scale. The Wright Item Map shows the hierarchical structure of the scale with a moderate degree of inter-item spread. And the standardized *t*-tests indicate a moderate degree of item overlap. Rasch analysis gives specific measurement properties that provide criterion for successful measurement and gives information regarding how well the criterion under consideration is met. Rasch analysis and factor analysis are complementary analytical frameworks. Both are used to evaluate the dimensionality of scales—that is, in identifying the number of traits (in this case behaviors) influencing the response patterns. Although factor analysis is commonly used to identify multiple sources of variation, it can indicate the degree of unidimensionality of a scale [27] which it has done in this instance. Since the Rasch analysis shows that the scale was largely unidimensional, it is not surprising that the factor analysis results have yielded a three factor solution heavily loaded on one factor. While the three factor solution presented here indicates that this scale has identifiable traits, the loadings should not be considered subscales in their own right. The factors are face valid but the items in the scale were carefully chosen to represent a single construct.

These results tie the scale to the social support literature and thus to social learning theory. From that theoretical perspective, the three peer focused factors: positive versus negative social behavior, drug use and post treatment peer association are consistent with Mason’s [19] discussion of the five network characteristics that are associated with adolescent substance use or non-use. This analysis expands Mason’s work in that it behaviorally defines those network characteristics and further demonstrates those behaviors association with adolescent substance use or non-use. These findings suggest that while this scale’s explanatory power is clear and it has significant potential as a clinical and research variable, the broader area of social support as it relates to this treatment population is certainly a fertile area for further research which would involve the development of companion scales.

## Conclusions

It is clear that understanding peer relations is of great importance in adolescent substance abuse treatment. Indeed a thorough understanding of the way adolescents give and receive social support—the role such support plays in the arousal persistence and direction of their behavior related to drug use, recovery from treatment and other aspects of SUD—is vital to the development of effective treatment modalities for adolescents with SUD. Given the research evidence and anecdotal knowledge that social support figures in prominently in so many aspects of adolescent substance use disorder, it is surprising that a standard measure of social support (or more specifically peer-related social support) has not emerged in the literature. Measures such as the one presented in this paper can serve as a research variable and as a standard measures of treatment success, but it is up to the substance abuse treatment and research communities to further investigate the relationship of peer relations and social support to the design, implementation, and evaluation of treatment strategies.

It is ideal for adolescents treated for SUD to refrain completely from interacting with friends who use drugs and alcohol and to associate with friends who engage in more positive behaviors. This scale is of value to the treatment community because of its brevity, clarity, and its emphasis on specific observable behaviors. Since the 14 items are linked to specific behaviors, the scale can be used during the frequent short follow-ups that are commonly part of relapse prevention programs. It will make the need for intervention for adolescents at high-risk or relapse apparent in time to make appropriate referrals.

While this research reveals program-level policy implications, one cannot help but think that there are broader policy implications stemming from the ability to operationalize and measure peer support. With measures such as this, treatment providers may be better able to design and enhance programming options. Policies as they relate to therapeutic communities and other support group intensive approaches to treatment can be enhanced because they will be better able to screen, track, and measure outcomes of this care. Simply put, better measures of peer support can make it easier to design more effective adolescent substance treatment programs as well as evaluate them.

## Abbreviations

DSM-IV, Diagnostic and Statistical Manual of Mental Disorders fourth edition; ASAM, American Society of Addiction Medicine; PPC-II, Patient Placement Criteria second edition; PCA, Principle Components Analysis; SUD, Substance Use Disorder.

## Competing interests

The authors have no competing interest or financial relationship with other people or organizations that would influence the analysis of data or presentation of information presented in this article.

## Authors’ contribution

JC and SS are the principal investigators of the study in which the data presented here were collected. They conceived of, designed, and are responsible for and participated in carrying out all aspects of the study. PY participated in the design of this study, did the statistical analysis and helped draft the manuscript. KM participated in the design of the study, offered theoretical guidance and helped draft the manuscript. All authors read and approved the final manuscript.
